# Sleep, rest-activity rhythm, cognitive and emotional symptoms in adult ADHD: unraveling the links with an actimetry-based approach

**DOI:** 10.1186/s12888-026-07947-9

**Published:** 2026-03-09

**Authors:** Amélia Walter, Émilie Martz, Luisa Weiner, Henri Comtet, Raphaëlle Glacet, Ülker Kilic-Huck, Patrice Bourgin, Carmen M. Schröder, Sébastien Weibel

**Affiliations:** 1 Institute of Cellular and Integrative Neurosciences, CNRS UPR 3212, University of Strasbourg, Strasbourg, France; 2 Sleep Disorders Centre & International Research Centre for ChronoSomnology (CIRCSom), Strasbourg University Hospital, Strasbourg, France; 3 Laboratory of Psychology of Cognitions, University of Strasbourg, Strasbourg, France; 4 Department of Psychiatry, Strasbourg University Hospital, Strasbourg, France; 5 Department of Child and Adolescent Psychiatry, Strasbourg University Hospital, Strasbourg, France; 6 UMR-S 1329, Strasbourg Translational Neuroscience & Psychiatry, INSERM, University of Strasbourg, Strasbourg, France

**Keywords:** Adult ADHD, Sleep, Actigraphy, Cognitive functions, Emotion dysregulation

## Abstract

**Background:**

Attention Deficit Hyperactivity Disorder (ADHD) in adults is characterized by cognitive, behavioral and affective symptoms. Emotion dysregulation (ED), psychiatric comorbidities, and disturbances in sleep and circadian rhythms frequently co-occur with ADHD, yet their relationships have rarely been investigated using objective measures and are overlooked in therapeutic approaches. This study aimed to compare sleep and rest–activity rhythm parameters between adults with ADHD and control subjects and to examine their associations with cognitive and emotional symptoms in adults with ADHD.

**Methods:**

Fifty-four adults with ADHD completed self-report questionnaires assessing inattention, hyperactivity/impulsivity, and emotional symptoms of ADHD, along with a neuropsychological evaluation of attentional and executive functions. Sleep patterns and rest-activity rhythm were monitored using actigraphy over a 10-days period and compared to data from 47 control participants without psychiatric or neurodevelopmental disorders, adjusting for age and sex. Relationships between ADHD symptoms, neuropsychological test results, sleep and rest–activity rhythm parameters were examined using correlation analyses.

**Results:**

Adults with ADHD exhibited a more unstable and delayed rhythm compared to controls. Sleep and rest–activity rhythm disturbances were significantly associated with cognitive and behavioral symptoms, particularly hyperactivity. Longer sleep latency was associated with poorer selective attention, while shorter total sleep time was linked to deficits in inhibitory control. However, no significant associations were found between sleep, circadian rhythms, and ED symptoms and the majority of observed correlations were no longer significant after correction for multiple comparisons.

**Conclusions:**

These findings highlight the intricate interplay between sleep and circadian rhythm disturbances and the core symptoms of ADHD, emphasizing the need to consider sleep and circadian rhythms interventions in the management of core ADHD symptoms in adults.

**Trial registration:**

The study involving adults with ADHD, Emotional Dysregulation and Cyclothymia in Adult Patients With ADHD (EMO-TDA), was registered at ClinicalTrials.gov (Identifier: NCT03494478) on April 11, 2018. The study involving control participants, Influence of Light on Sleep, Awakening, Electroencephalogram (EEG) and Cognitive Performances, and Medical Technology Assessment for Registration and Long-term EEG Analysis (CHRONOSOMNO), was registered at ClinicalTrials.gov (Identifier: NCT02858765) on August 8, 2016.

**Supplementary Information:**

The online version contains supplementary material available at 10.1186/s12888-026-07947-9.

## Introduction

Attention Deficit Hyperactivity Disorder (ADHD) is a prevalent neurodevelopmental disorder characterized by persistent symptoms of inattention, hyperactivity and impulsivity [1]. While commonly diagnosed in childhood, ADHD persists in adulthood in approximately two-thirds of cases affecting 2.6% to 6.8% of adults worldwide [2]. Interestingly, the symptomatology in adults evolves, and as an example, hyperactivity tends to be internalized resulting in a subjective acceleration and overproduction of thoughts, i.e., racing thoughts [3]. The quality of life of adults with ADHD is also frequently impacted by neurodevelopmental and psychiatric comorbidities [4], as well as emotional difficulties [5, 6]. Indeed, additional symptoms of emotion dysregulation (ED) such as lack of emotional control, emotional lability and over-reactivity are reported in 30 to 70% of cases. Additionally, sleep difficulties and alterations of rest–activity rhythms affect 60–80% of adults with ADHD, further impairing their quality of life [7].

Although much of the research on sleep and circadian rhythm has been conducted in childhood ADHD [8], sleep problems in adults with ADHD can have serious consequences such as an increased risk of driving accidents [9], chronic health conditions [10], and exacerbation of psychiatric comorbidities [11]. One explanation is related to the fact that sleep difficulties can affect emotion regulation and mood in both clinical and non-clinical populations [12]. Indeed, Rapid Eye Movement (REM) sleep contributes to emotion regulation, by dampening the emotional intensity associated with life events [13]. At a neurobiological level, sleep debt leads to reduced connectivity between regions involved in emotion regulation [14]. From a behavioral standpoint, it heightens emotional reactivity and impulsivity and weakens emotion monitoring [15]. Therefore, understanding the specificities of sleep problems in adult ADHD is crucial.

The etiology of sleep difficulties in ADHD is likely to be multifactorial. Sleep problems can be explained by delayed circadian rhythms [16] affecting up to 78% of adults with ADHD [17] which is associated with evening preference or evening chronotype, difficulties falling asleep at night, and waking up in the morning at socially imposed times earlier than their endogenous circadian preference, and diurnal sleepiness [7, 18, 19]. Moreover, poor sleep hygiene, psychiatric comorbidities, ED, racing thoughts and rumination may contribute to the development and maintenance of sleep difficulties and rest–activity rhythms misalignment in ADHD [3, 20, 21]. For instance, motor hyperactivity and racing thoughts in the evening and at bedtime have been associated to sleep problems [3]. Interestingly, sleep difficulties may vary depending on the specific presentation of ADHD in children, with circadian rhythm problems, sleep-talking and nightmares being mainly associated with the combined presentation, while hypersomnia is associated with the inattentive presentation [22]. Gamble et al. [23] also found that delayed sleep onset and daytime sleepiness were associated with an increased severity of inattention, hyperactivity and impulsivity in adult ADHD.

Regarding emotional symptoms, Syrstad et al. [24] found that cyclothymic temperament is associated with sleep-wake rhythm instability in adults with ADHD. Moreover, individuals with delayed sleep–wake patterns tend to go to bed later and may extend their sleep duration on free days, leading to shifts in the timing and distribution of REM sleep. REM sleep follows a circadian rhythm, with higher proportions occurring in the early morning [25, 26], suggesting that chronic misalignment of sleep timing during the week could particularly reduce REM sleep—an effect that is especially relevant given REM’s role in emotion regulation.

From a neuropsychological standpoint, daytime sleepiness has been shown to impact cognitive performances, especially attention tasks, as demonstrated in adults with ADHD [27] and may further exacerbate the neurobiological attentional deficits inherent to ADHD [28]. Sleep instability and circadian phase shift, particularly in shift workers, have been linked to impairments of memory, attention and executive functions [29].

Despite the intuitive links between sleep problems and cognitive and emotional symptoms in adult ADHD, only few studies have attempted to disentangle these mutual relationships in this age group. Dolapoglu et al. [30] highlighted an interaction between ED and sleep quality, as measured by the total scale as well as the sleep latency and sleep duration subscales of the Pittsburgh Sleep Quality Index (PSQI) [31], a widely used self-report questionnaire assessing subjective sleep quality. Moreover, Helfer et al. [27] found that mindwandering (i.e., the shift of attention away from a primary task toward internal and task-unrelated thoughts), emotional lability, and poor sleep quality are interrelated and together significantly contribute to the severity of ADHD symptoms in adults. However, the directionality of these associations remains unclear. While sleep difficulties can exacerbate cognitive and emotional difficulties, the consequences of cognitive and ED symptoms (e.g., irritability, and angry outbursts) may in turn lead to sleep problems, further contributing to emotional and cognitive impairment [27]. Despite their prevalence and impact, sleep problems are often overlooked and left untreated.

Additionally, studies investigating the links between affective functioning, sleep and circadian rhythms in adult ADHD have relied almost exclusively on subjective measures, which may suffer from several biases (e.g., recall bias; [32]). However, most studies using objective measures such as polysomnography (PSG) have failed to establish consistent differences between participants with ADHD and neurotypical controls [33–35]. This inconsistency may be related to the high intra-individual variability in their day-to-day sleep-wake rhythms, which single or twice night PSG studies may fail to capture. Moreover, PSG assessments are often limited by inter-scorer variability [36]. Actigraphy, a validated ecological assessment tool, provides an alternative by monitoring sleep-wake patterns over extended periods, allowing the dynamic and complex nature of sleep to be captured. The utility of actigraphy has previously been validated in the field of neurodevelopmental disorders in children [37]. Nevertheless, the number of studies using actigraphy in adults with ADHD remains minimal compared to studies in children, and none have studied the links between sleep, circadian parameters and ED [38]. In terms of actigraphy-derived sleep parameters, there is a consensus that adults with ADHD experience a longer sleep onset latency and lower sleep efficiency compared to control subjects [17, 18, 39]. Regarding circadian parameters, discrepant results have been reported. Some studies are supportive of rhythm instability, variability and shifting in adults with ADHD [23], while others suggest a more stable and less variable rhythm compared to control individuals [17, 18, 40] or observe no differences at all [39, 41].

This study aims to investigate the relationships between sleep, circadian rhythm, cognitive and emotional symptoms in adults with ADHD, using both subjective measures (i.e., self-reported questionnaires) and objective measures (i.e., neuropsychological assessments and actigraphy recordings) compared to neurotypical subjects. Our first aim is to characterize the sleep and circadian rhythm patterns of adults with ADHD compared to a group of neurotypical controls. We expect that adults with ADHD will exhibit longer sleep onset latency and lower sleep efficiency, particularly in the combined presentation, where hyperactivity and impulsivity may exacerbate sleep disruptions. Regarding circadian rhythms, despite conflicting findings in the literature, adults with ADHD are expected to exhibit more instability and rhythmic shifts, reflecting an evening chronotype. We further hypothesize that these irregular sleep–wake patterns in adults with ADHD, potentially causing weekday sleep restriction, may contribute to greater hyperactivity and ED. In addition, inattention and hyperactivity symptoms are expected to correlate strongly with sleep latency, potentially driven by racing thoughts at bedtime. In an exploratory way, we aim to measure the effect of ED and psychiatric comorbidities (i.e., anxiety, depression) on sleep and circadian rhythm. We hypothesize a link between emotional instability and the instability of the sleep/wake rhythm.

## Methods

### Participants

#### ADHD group

Fifty-four newly diagnosed adults with ADHD aged 18–57 years (*M* = 32.5; *SD* = 9.78) were recruited at the University Hospitals of Strasbourg (Table 1) . The diagnostic interview was led by senior psychiatrists according to DSM-5 criteria for ADHD [1]. To ensure a representative sample of the group of adults with ADHD and to assess the effect of comorbidities, we did not exclude subjects with psychiatric and neurodevelopmental comorbidities. As the patients had been recently diagnosed, no psychostimulant treatment was initiated before the cognitive evaluation and actigraphy recording. However, ongoing treatments for comorbidities were allowed during the study. This study is part of a large project on ED in ADHD, which received ethical approval from CPP South Mediterranean II (IDRCB: 2017-A01618-45 and CPP East of France; No. SI 21.01.21.41923).

#### Control group

Forty-seven control subjects aged 18–36 years (*M* = 23.3; *SD* = 3.66) were recruited as part of a larger chronobiological protocol within the University Hospital of Strasbourg which was also approved by the ethical committee (CPP Est IV n°14/29). Recruitment of participants was mainly done though the hospital network. Neurotypical controls underwent a psychiatric assessment using the Mini International Neuropsychiatric Interview [42] and completed self-reported questionnaires to ensure the absence of psychiatric or neurodevelopmental disorders, as well as sleep disorders or alterations of rest–activity rhythms.

### Materials and procedures

#### Questionnaires

To assess symptoms related to sleep-wake rhythms and sleep disorders, we used the Epworth Sleepiness Scale (ESS) [43], the Insomnia Severity Index (ISS) [44], a list of DSM-IV criteria of restless legs syndrome (RLS), and obstructive sleep apnea (OSA) [45]. The Morningness-Eveningness Questionnaire (MEQ) [46] was used to assess chronotype (i.e., an individual’s stable preference for the timing of sleep and activity).

ADHD symptoms were screened using the Wender-Reimherr Adult Attention Deficit Disorder Scale (WRAADDS) which is a self-reported scale assessing the three core symptoms of ADHD (i.e., inattention, hyperactivity and impulsivity) and some dimensions related to ED (e.g., affective lability and emotional over-reactivity) [47]. We also measured emotional lability using the Affective Lability Scale (ALS) [48]. The Racing and Crowded Thoughts Questionnaire (RCTQ) [49] assessed frequency of racing thoughts at different times of day, including morning, afternoon, evening, and bedtime.

Psychiatric and neurodevelopmental comorbidities were screened by the Beck Depression Inventory (BDI) [50], the Generalized Anxiety Disorder assessment 7-item (GAD-7) [51] and the 10-item Autism Spectrum Quotient (AQ-10) [52]. Notably, comorbidities with autism were assessed due to the high comorbidity with ADHD (i.e., 50–70% of autistic individuals also meet criteria for ADHD; [53]) and the overlap in sleep difficulties observed in both conditions [54].

#### Neuropsychological assessment

The neuropsychological assessment focused on executive and attentional functions. Verbal working memory was assessed using the Digit Span subtest of the WAIS-III (Wechsler Intelligence Scale for Adults − 3rd edition) [55]. To investigate inhibitory functions, subjects performed: the Go-Nogo subtest of the TAP 2.3 (1-target and 2-target) [56] assessing motor inhibition, and the Hayling test [57] assessing verbal response inhibition were administered. Inhibition cost was defined as the difference between the mean response time in the inhibition condition and that in the automatic condition. The Trail Making Test (TMT) [58] was administered to measure attentional switching. Verbal initiation and spontaneous flexibility were assessed via the Verbal Fluency Test [59], under both categorical and phonological conditions. The Tower of London test [60] was used to assess planning strategies. The TAP 2.3 subtests “Sustained Attention” (colors and shapes), and “Divided Attention” (dual-task) and the Ruff 2 and 7 selective attention test [61] were used to assess attention abilities.

#### Actigraphy

In the ADHD study, actigraphic data were obtained using an ActiGraph wGT3X-BT worn on the nondominant wrist for ten days and nights. Control subjects wore the ActiGraph wGT3X-BT or the MotionWatch for 6 to 22 days and nights. The data were respectively processed on the ActiLife software © (version 6.7) with the Cole-Kripke sleep scoring algorithm and the MotionWare software © (version 1.2.47). Notably, to account for potential differences in movement sensitivity between the two devices, we compared the sleep parameters of interest between the two watches in another protocol led in our lab. In this protocol, eight subjects wore simultaneously the ActiGraph and MotionWatch devices during three consecutive days. No differences were found for sleep latency, Total Sleep Time (TST), wake-up after sleep onset (WASO), and sleep efficiency. However, the number of awakenings and the average duration of awakenings differed between the two watches, hence we did not investigate these parameters when comparing the two groups (see Supplementary material A).

**Table 1:** Demographic, sleep and circadian rhythm characteristics of participants

	ADHD group (*n* = 53)	Control group (*n* = 47)	*p*-value	*p*-value after adjustment for age and sex	Effect size (η²*p* and ε²)
**Demographic characteristics**					
Age^a^	32.50 (9.78)	23.3 (3.66)	< 0.001**	-	-
Sex	*n* = 31 W, 22 M	*n* = 26 W, 21 M	0.749	-	-
SP status: disability	*n* = 1	*n* = 0	-	-	-
SP status: seeking employment	*n* = 14	*n* = 2			
SP status: student	*n* = 11	*n* = 36	-	-	-
SP status: full-time or part-time worker	*n* = 27	*n* = 8	-	-	-
**ADHD presentation**,** comorbidities and treatments**					
ADHD presentation	*n* = 43 combined, *n* = 10 inattentive	-	-	-	-
Autism Spectrum Disorder	*n* = 4	-	-	-	-
Anxiety Disorder	*n* = 4	-	-	-	-
Borderline Personality Disorder	*n* = 4	-	-	-	-
Bipolar Disorder	*n* = 6	-	-	-	-
Cyclothymia	*n* = 4	-	-	-	-
Antidepressants	*n* = 8	-	-	-	-
Anxiolytics	*n* = 4	-	-	-	-
Mood Stabilizers	*n* = 4	-	-	-	-
**Sleep and circadian questionnaires**					
Restless Legs Syndrome	2.23 (1.40)	-	-	-	-
Sleep Apnea Syndrome	1.17 (0.97)	-	-	-	-
Epworth Sleepiness Scale^a^	10.2 (5.74)	4.28 (2.73)	< 0.001***	0.002**	0.100
Insomnia Severity Index^a^	13.1 (5.49)	1.84 (2.10)	< 0.001***	< 0.001***	0.446
Morningness-Eveningness Questionnaire^a^	46.3 (13.5)	56.2 (7.16)	< 0.001***	< 0.001***	0.222
**Sleep and circadian rhythm characteristics**					
Sleep latency (min)^a^	9.10 (4.13)	8.89 (8.00)	0.536	0.727	0.000
TST (min)	448 (57.5)	438 (38.8)	0.310	0.328	0.010
TST weekdays (min)^a^	447 (65.4)	428 (79.7)	0.506	0.867	0.000
Sleep efficiency^a^	87.8 (4.74)	87.5 (4.98)	0.989	0.691	0.000
WASO (sum of awakenings ≥ 1 min)	54.4 (24.0)	52.2 (21.3)	0.485	0.301	0.011
Inter-day stability (IS)	0.450 (0.125)	0.488 (0.140)	0.169	0.029*	0.053
Intra-day variability (IV)	0.883 (0.212)	0.864 (0.261)	0.689	0.502	0.005
Start of most active phase (M10)^a^	10 h 40 (2 h 20)	10 h 06 (1 h 33)	0.227	**0.054**	0.040
Start of least active phase (L5)^a^	1 h 58 (2 h 01)	0 h 51 (1 h 01)	0.001**	< 0.001***	0.173

Data are presented as n or mean (SD)

p-values in bold < 0.10; *p < 0.05; **p < 0.01; ***p < 0.001

^a^Non-parametric tests applied to non-normal or non-homogeneous data

SP= Socio-professional; TST = Total Sleep Time; WASO = Wake-up After Sleep Onset

Sleep efficiency is calculated by dividing the amount of time spent asleep by the total amount of time in bed, while the WASO represents the amount of time a person spends awake after sleep onset. In this study, nocturnal awakenings in adults with ADHD were categorized by duration (i.e., exceeding one minute, five minutes, or ten minutes) to differentiate micro-awakenings from longer awakenings, which are more disruptive to sleep efficiency. Additionally, participants completed a daily sleep diary to determine sleep and wake-up times. However, due to frequent omissions in diary completion, we chose to estimate bedtime and wake-up times based on objective data (i.e., the changes in activity levels and lights-off) obtained from actigraphy.

Actigraphy data were analyzed using non-parametric circadian rhythm analysis (NPCRA) to provide insights into different aspects of daily activity and rest patterns. For the ActiGraph data, analyses were performed using the actiCircadian program [62]. These measures included intra-day variability (IV), inter-day stability (IS), M10 onset and L5 onset, each of which reflects a specific component of circadian regulation [63]:

- Intra-day variability (IV) quantifies the degree of fragmentation in activity and rest periods throughout the day. This measure is particularly relevant for identifying irregular patterns, such as frequent transitions between motor activity (e.g., wakefulness, nocturnal agitation) and inactivity (e.g., sleep, naps, lack of daytime activity). Higher IV values indicate greater fragmentation, which may reflect disrupted or erratic daily rhythms, with values ranging from 0 to 2.
- Inter-day stability (IS) assesses the regularity of activity patterns across different days. This metric captures the extent to which daily rhythms are stable, where 0 indicates no rhythm and 1 corresponds to a more regular and predictable routine.
- M10 onset, the median start time of the 10 most active consecutive hours, highlights the core period of sustained daily activity. This measure provides insight into the timing of peak activity, offering a marker of diurnal preference or showing shifts in the active phase of the circadian cycle.
- L5 onset, the median start time of the 5 least active consecutive hours, represents the core period of inactivity or rest. This measure is critical for understanding rest patterns and sleep timing. Delayed or advanced L5 onset may indicate phase shift [64, 65].

### Statistical analyses

One-way ANOVA and post hoc Tukey were conducted to compare demographic, sleep, circadian rhythm, psychopathological, and cognitive characteristics of participants between groups. When assumptions for parametric tests were not met, Kruskal-Wallis and Mann-Whitney U tests were used. Given the significant age difference between participant groups, as well as the well-documented influence of age and sex on sleep and circadian rhythm parameters [66, 67], analyses of covariance (ANCOVA) were performed with age and sex assigned at birth (categorized as women or men) as covariates. Effect sizes were estimated using partial eta-squared (*η²p*) for ANCOVA analyses. For non-parametric tests, epsilon-squared (ε²) was calculated for Kruskal-Wallis tests, and rank-biserial correlations were computed for Mann-Whitney U tests, to quantify the magnitude of group differences.

In an exploratory approach, associations between ADHD symptoms, neuropsychological test results, sleep, and circadian parameters were examined using Pearson’s correlation coefficient. Spearman correlations were performed for non-normal and non-homogeneous data. Normality and homogeneity were verified using the Shapiro-Wilk and Levene tests, respectively. Equivalence between sleep characteristics obtained with the MotionWatch and the ActiGraph device was tested using the two one-sided test (TOST).

As these were exploratory analyses, no initial correction for multiple comparisons was applied. However, in a second step, to account for multiple testing correlation analyses, the false discovery rate (FDR) correction method was applied [68], which adjusts p-values based on their rank among all tests. This approach is less conservative than the Bonferroni correction, allowing control of type I errors while maintaining greater statistical power.

Finally, to investigate the influence of sleep quality on psychopathological and cognitive characteristics, participants were categorized into three groups based on actigraphic-measured sleep efficiency to better identify non-linear effects and highlight extreme sleep profiles. Indeed, sleep efficiency reflects both sleep onset difficulties and nighttime awakenings, providing a global index of sleep quality. Those with a score below the first quartile (≤ 85%) were classified as the low efficiency group (*n* = 13), those above the third quartile (≥ 90%) as the high efficiency group (*n* = 15), and those between 85% and 90% as the median efficiency group (*n* = 23).

## Results

### Comparison of demographic, sleep and circadian rhythm characteristics between adults with ADHD and control subjects

Comparison between adults with ADHD and control subjects (Table 1) indicates that adults with ADHD had a significantly higher mean age compared to the control group (*M* = 32.5 years vs. *M* = 23.3 years; *p* < 0.001).

Among the 53 adults with ADHD, 43 had a combined presentation of ADHD, and 10 had the inattentive presentation. In terms of comorbidities, 4 participants also had autism, 4 had anxiety disorders, 4 showed symptoms of borderline personality disorder, 6 were diagnosed with bipolar disorder, and 4 with cyclothymia. As for medication use, 8 participants were treated with antidepressants, 4 were taking anxiolytics, and 4 were on mood stabilizers. None of the control subjects had any comorbidities or were taking medication.

Regarding sleep and circadian rhythm characteristics, ADHD participants exhibited higher levels of daytime sleepiness (*M* = 10.2 in the ADHD group vs. *M* = 4.28 in controls; *p* = 0.002, *ε²* = 0.100), greater insomnia severity (*M* = 13.1 vs. *M* = 1.84; *p* < 0.001, *ε²* = 0.446) and greater eveningness in their chronotypes (*M* = 46.3 vs. *M* = 56.2; *p* < 0.001, *ε²* = 0.222) compared to controls. Moreover, the onset of the rest-phase period (L5) is also delayed in this group, even after adjusting results for age and sex group (χ² = 15.7; *p* < 0.001, *ε*² = 0.173).

After controlling for age and sex differences, adults with ADHD showed lower rhythm stability (IS) across days compared to control subjects (*M* = 0.450 in the ADHD group vs. *M* = 0.488 in controls; *F* = 4.95; *p* = 0.029, *η²p* = 0.053), indicating a moderate effect size. In addition, these analyses revealed an age and sex-related trend difference in start times of the active phase (M10) (*χ²* = 3.70; *p* = 0.054, *ε²* = 0.040). No differences were found for other actigraphic characteristics (*p* > 0.05).

Regarding the differences in sleep parameters and rhythm between the inattentive presentation ADHD group and the combined presentation, there were non-significant trends in WASO (Supplementary material B), specifically for the total wake time due to arousals lasting longer than 1 min (*p* = 0.088; *ε²* = 0.061) and 5 min (*p* = 0.056; *ε²* = 0.074). Notably, adults with the combined presentation tended to spend more time awake after sleep onset (WASO ≥ 5 min; *M* = 35.9 min) compared to those with the inattentive presentation (*M* = 23.8 min).

Within the ADHD group, IS was positively associated with total sleep time (*r* = 0.311, *p* = 0.033, Table 2) and sleep latency (*ρ* = 0.364, *p* = 0.012). IV was negatively associated with the number of awakenings (*r* = − 0.294, *p* = 0.042), while later timing of M10 onset (*ρ* = 0.350, *p* = 0.015) and L5 onset (*ρ* = 0.317, *p* = 0.028) were both associated with a higher number of awakenings.

**Table 2:** Correlation analyses between sleep and circadian rhythm characteristics (ADHD group, *n* = 54)

	TST	TST weekdays	Sleep latency	Sleep efficiency	WASO	Number of awakenings	M10 (counts)	L5 (counts)
IS	0.311*	0.222	**0.364 ** ^S*^	− 0.136 ^S^	0.102	0.143	**0.427 ** ^**S**^ **	**-0.396 ** ^**S**^ **
IV	− 0.111	− 0.078	− 0.220 ^S^	0.066 ^S^	− 0.124	− 0.294*	**-0.637** ^S^ ***	-0.083 ^S^
M10 onset	0.036 ^S^	0.127 ^S^	− 0.023 ^S^	− 0.129 ^S^	0.155 ^S^	**0.350** ^S*^	**-0.338** ^S*^	**0.269** ^**S**^
L5 onset	0.126 ^S^	.161^S^	0.065 ^S^	− 0.050 ^S^	0.139 ^S^	**0.317** ^S*^	**-0.255** ^**S**^	**0.313** ^S^
M10 (counts)	-0.128 ^S^	-0.104 ^S^	0.152 ^S^	-0.044 ^S^	-0.032 ^S^	0.066 ^S^	/	/
L5 (counts)	**-0.412** ^S^**	**-0.354** ^S*^	-0.184 ^S^	**-0.336** ^S*^	**0.274** ^**S**^	**0.260** ^**S**^	0.046 ^S^	**/**

p-values in bold < 0.10; *p < 0.05; **p < 0.01, ***p < 0.001)

^S^Non-parametric test (Spearman correlation coefficient) applied to non-normal data

TST = Total Sleep Time; WASO = Wake-up After Sleep Onset; IS = Inter-day stability; IV = Intra-day variability; M10 onset = Start of most active phase; L5 onset = Start of least active phase

Greater activity during the most active 10 h (M10) was associated with more stable inter-day rhythms (*ρ* = 0.427, *p* = 0.003), reduced intra-day variability (*ρ* = -0.637, *p* < 0.001), and earlier timing of peak activity (*ρ* = -0.338, *p* = 0.018). In contrast, elevated activity during the least active 5 h (L5) was linked to less stable rhythms (*ρ* = -0.396, *p* = 0.006), reduced total sleep time (*ρ* = -0.412, *p* = 0.004; weekdays: *ρ* = -0.354, *p* = 0.015), and lower sleep efficiency (*ρ* = -0.336, *p* = 0.019). Using the false discovery rate correction, only the association between M10 activity and intra-day variability (IV) remained significant (*ρ* = −0.637, *p*_FDR = 0.045), whereas all other associations did not survive FDR correction (*p* > 0.05).

**Fig. 1:**
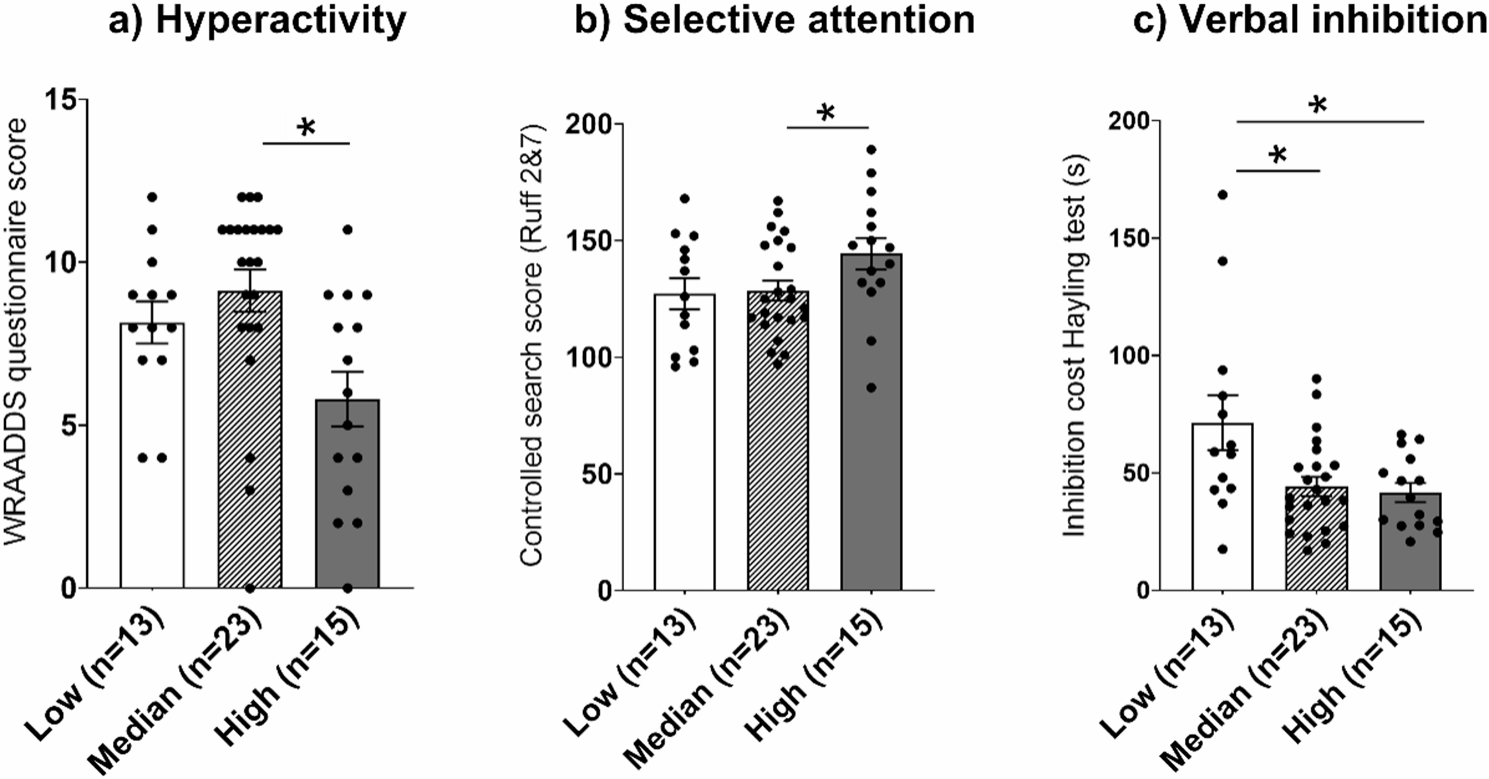
Behavioral and cognitive characteristics according to sleep efficiency profiles: (**a**) Self-reported hyperactivity (WRAADDS sub-scale) according to participants’ sleep efficiency. (**b**) Selective attention score (Ruff 2 & 7 test), with higher scores indicating better target detection and faster selective attention speed, according to participants’ sleep efficiency. (**c) **Time required to inhibit the verbal response in the Hayling test (i.e., inhibition cost, defined as the difference between the mean response times in the inhibition and automatic conditions) across sleep efficiency groups. *p < 0.05

In terms of behavioral and cognitive characteristics, adults with ADHD with higher sleep efficiency report fewer symptoms of hyperactivity (*M* = 5.80) compared to subjects with median sleep efficiency (*M* = 9.13; *p* = 0.002; *r* = 0.597, Fig. 1a), as well as better selective attention speed compared to the median group (*M* = 144 vs. *M* = 129; *p* = 0.041; *r* = 0.400, Fig. 1b). Subjects with low sleep efficiency show longer response times to inhibit the verbal response in the Hayling test (i.e., inhibition cost, indicative of reduced verbal inhibition abilities) compared to the median and high sleep efficiency groups (*M* = 71,5 vs. *M* = 44,3 and *M* = 41,7; *p* = 0.031; *r* = 0.472 and *p* = 0.033; *r* = 0.477, respectively, Fig. 1c).

### Correlation analyses between sleep, circadian rhythm, ADHD symptoms and cognitive symptoms (ADHD group)

Self-reported hyperactivity in adults with ADHD was negatively correlated with sleep efficiency (*ρ* = -0.314; *p* = 0.023; Table 3) and positively correlated with wake time after sleep onset (wake episodes > 5 min; *ρ* = 0.317; *p* = 0.021; Table 3). However, no significant association was found between hyperactivity and circadian parameters (*p* > 0.05). Similarly, inattention and impulsivity were not significantly related to sleep or circadian measures (*p* > 0.05).

**Table 3:** Correlation analyses between sleep, circadian rhythm, ADHD symptoms, anxiety and depression symptoms (ADHD group, *n* = 54)

	Inattention (WRAADDS)	Hyperactivity (WRAADDS)	Impulsivity (WRAADDS)	Emotional hyperreactivity (WRAADDS)	Affective lability (WRAADDS)	Cyclothymic temperament (TEMPS)	Anxiety (GAD)	Depression (BDI)	Racing thoughts (RCTQ)	Racing thoughts at bed time (RCTQ)
TST	− 0.097	− 0.025 ^S^	− 0.004	**0.361 ** ^**S**^ **	0.113 ^S^	0.054 ^S^	0.080	0.229	**− 0.281**	− 0.086
TST weekdays	− 0.119	0.035 ^S^	− 0.092	**0.301** ^S^*	0.032 ^S^	0.637 ^S^	0.017	0.194	− 0.250	− 0.069
Sleep latency	− 0.061 ^**S**^	0.254 ^S^	0.098 ^S^	0.086 ^S^	0.033 ^S^	0.120 ^S^	0.145 ^S^	0.186 ^S^	0.087 ^S^	0.099 ^S^
Sleep efficiency	− 0.046 ^**S**^	**− 0.314** ^S^*	− 0.142 ^S^	− 0.022 ^S^	− 0.093 ^S^	− 0.089 ^S^	− 0.112 ^S^	− 0.128 ^S^	− 0.176 ^S^	− 0.096 ^S^
WASO (sum of awakenings ≥ 5 min)	0.075	**0.317** ^S^*	0.169 ^S^	0.121 ^S^	0.038 ^S^	0.024 ^S^	0.218	0.221	0.224	0.166
Number of awakenings (> 10 min)	0.014	**0.322** ^S^*	0.146 ^S^	0.091 ^S^	0.049 ^S^	0.072 ^S^	0.236 ^S^	− 0.138 ^S^	0.322*	0.226
IS	− 0.125	0.059 ^S^	0.006	0.046 ^S^	0.014 ^S^	− 0.037 ^S^	0.007	0.080	− 0.141	− 0.203
IV	0.172	− 0.236 ^S^	0.032	− 0.016 ^S^	0.122 ^S^	0.064 ^S^	0.019	0.041	0.074	− 0.013
M10	− 0.046 ^S^	0.104 ^S^	0.032 ^S^	− 0.155 ^S^	0.124 ^S^	0.053 ^S^	− 0.175 ^S^	− 0.151 ^S^	− 0.011 ^S^	− 0.067 ^S^
L5	− 0.030 ^S^	0.037 ^S^	− 0.050 ^S^	0.075 ^S^	0.196 ^S^	0.006 ^S^	− 0.030 ^S^	0.062 ^S^	0.078 ^S^	0.014 ^S^

p-values in bold < 0.10; *p < 0.05; **p < 0.01

^S^Non-parametric test (Spearman correlation coefficient) applied to non-normal data

WRAADDS = Wender-Reimherr Adult Attention Deficit Disorder Scale; GAD = Generalized Anxiety Disorder assessment; BDI = Beck Depression Inventory; RCTQ = Racing and Crowded Thoughts Questionnaire; TST = Total Sleep Time; WASO = Wake-up After Sleep Onset; IS = Inter-day stability; IV = Intra-day variability; M10 = Start of most active phase; L5 = Start of least active phase

With regard to psychopathological symptoms, a positive correlation emerged between the number of nocturnal awakenings (lasting more than 10 min) and self-reported symptoms of racing thoughts (*r* = 0.322, *p* = 0.020; Table 3). Additionally, total sleep time was positively associated with emotional hyperreactivity across the entire week (*ρ* = 0.361, *p* = 0.008; Table 3) and on weekdays only (*ρ* = 0.301, *p* = 0.030; Table 3). Autistic traits, anxiety and depression, were not associated with sleep and circadian rhythm parameters (*p* > 0.05).

Regarding attention measures, analyses revealed significant links with sleep latency: longer sleep latency in adults with ADHD was associated with lower scores on the selective attention test (*ρ* = -0.370, *p* = 0.007; Table 4). In terms of executive functions, total sleep time on weekdays was significantly associated with reaction time in the Go/No-Go task (*r* = 0.366, *p* = 0.008; Table 4) and Hayling test response time (*ρ* = -0.392, *p* = 0.005, *ρ* = -0.285, *p* = 0.041 for TST across the total week; Table 4). However, none of the correlations between sleep, circadian rhythm, ADHD symptoms and cognitive performance (Table 4) remained significant after the FDR correction (*p* > 0.05).

**Table 4:** Correlation analyses between sleep, circadian rhythm, attentional and executive functions (ADHD group, *n* = 54)

	TAP Sustained attention (RT)	TAP Sustained attention (RT variability)	TAP Divided auditory attention (correct answers)	TAP Divided visual attention (correct answers)	Ruff 2&7 Selective attention (score)	TAP Go-Nogo 1 (RT)	TAP Go-Nogo 2 (RT)	Digit span forward (number of digits)	Verbal fluency letter (number of words)	Verbal fluency semantic (number of words)	Stroop test -Interference minus Reading (time)	Hayling test - Inhibition minus Automatic (time)	TMT A (time)	TMT (B-A)/A (time)	Tower of London (initiation time)
TST	0.052	0.087	− 0.067 ^S^	− 0.008 ^S^	0.014	0.183	**0.238**	0.080 ^S^	0.086	− 0.053	− 0.009	**− 0.285 ** ^**S**^ *	− 0.035	.088^S^	**− 0.258 ** ^**S**^
TST weekdays	0.178	0.092	− .050^S^	.079^S^	0.065	0.268	0.366*	**− 0.290** ^**S**^ *	0.187	− 0.002	0.059	**− 0.392** ^**S**^ *	− 0.033	.192^S^	− .247^S^
Sleep latency	**− 0.289** ^S^ *	− .238^S^	− .002^S^	.012^S^	**− 0.370** ^**S**^ *	.103^S^	.101^S^	− .201^S^	.046^S^	.093^S^	.152^S^	.231^S^	0.098	− .174^S^	.091^S^
Sleep efficiency	.186^S^	.084^S^	− .135^S^	.025^S^	.239^s^	.112^S^	.038^S^	.126^S^	− .015^S^	− .175^S^	− .141^S^	− .239^S^	− 0.059	.025^S^	− .084^S^
WASO (≥ 5 min)	− 0.143	− 0.019	− .032^S^	− .032^S^	− 0.210	− 0.048	0.027	− .061^S^	− 0.045	0.115	0.087	.190^S^	0.020	.060^S^	.018^S^
Number of awakenings (> 10 min)	− .155^S^	− 0.076	− .006^S^	− .024^S^	− .270^S^	− .017^S^	.130^S^	− .054^S^	.079^S^	.121^S^	.002^S^	.101^S^	0.003	.212^S^	− .136^S^
IS	− 0.303*	− 0.271	.126^S^	− .007^S^	− 0.134	0.001	0.028	− .160^S^	- 0.064	0.077	0.190	.074^S^	− 0.147	− .079^S^	− .001^S^
IV	0.144	0.072	**− 0.292** ^S^*	− .026^S^	0.020	0.108	0.167	.072^S^	0.149	0.006	− 0.117	− .194^S^	− 0.060	.157^S^	− .029^S^
M10	.271^S^	0.172	− .139^S^	.013^S^	− .085^S^	.149^S^	.019^S^	− .195^S^	.098^S^	− .077^S^	.042^S^	− .112^S^	0.137	.103^S^	.032^S^
L5	.189^S^	− 0.010	− .147^S^	− .033^S^	− .050^S^	− .074^S^	- .167^S^	.039^S^	.134^S^	− .026^S^	− .264^S^	.002^S^	− 0.027	**0.282** ^**S**^	− .027^S^

p-values in bold < 0.10; *p < 0.05

^S^Non-parametric test (Spearman correlation coefficient) applied to non-normal data

TAP = Test of Attentional Performance; TMT = Trail Making Test; RT = Reaction Time; TST = Total Sleep Time; WASO = Wake-up After Sleep Onset; IS = Inter-day stability; IV = Intra-day variability; M10 = Start of most active phase; L5 = Start of least active phase

## Discussion

This study aimed to investigate sleep, rest-activity rhythms, cognition and ED in adults with ADHD. First, our results highlight an unstable rhythm with a phase shift in adult ADHD comparatively to controls, as reflected by reduced inter-day stability and a delayed L5 onset. This finding aligns with differences in subjective circadian preferences, as measured by the chronotype questionnaire, which revealed a more pronounced evening chronotype in adults with ADHD compared to neurotypical controls. This phase shift may be explained by the delayed melatonin secretion observed in ADHD [17] and/or by poor sleep hygiene. For instance, individuals with ADHD may be more easily distracted at nighttime by stimulating activities like video games or media use [69], leading to late light exposure that interferes with melatonin secretion. This increased distractibility in the evening may be further exacerbated by their phase-delayed circadian rhythm, which shifts their alertness peak into the pre-bedtime hours [70].

The greater day-to-day instability observed in adults with ADHD compared to control subjects, is also consistent with a previous study [23] studying comorbidity-free adults with ADHD over a two-week period. However, Boonstra et al. [18] found no differences in circadian parameters, suggesting that adults with ADHD may compensate for their instability by imposing an external rhythm [71]. Compensation may be less pronounced in younger people, such as those recruited in our study. Moreover, our adult ADHD group is two times larger than those from previous studies [17, 18, 39, 41], which may have increased the statistical power of our analyses. Importantly, differences in inter-day stability cannot be attributed to device differences, as this metric reflects the regularity of daily activity patterns rather than absolute activity levels, making it robust across devices.

Beyond these group-level comparisons, we explored whether irregular sleep–wake patterns could be linked to weekday sleep restriction—potentially including REM sleep—and thereby to greater hyperactivity and ED in adults with ADHD. While these associations were not consistently supported, several modest links emerged between circadian rhythm indices and sleep fragmentation. Greater IS was associated with longer total sleep time but also with longer sleep latency (Table 2), indicating that regular schedules promote longer sleep duration despite potential misalignment with immediate sleep propensity. Importantly, this may explain the absence of group differences in sleep latency: adults with ADHD, having less stable rhythms, may compensate by going to bed more flexibly when sleepiness occurs, facilitating sleep onset. In addition, a higher number of awakenings was associated with later timing of both M10 and L5 onsets, suggesting that fragmented sleep co-occurs with delayed sleep-wake timing. Interestingly, both higher daytime activity and more awakenings were related to lower IV. In adults with ADHD, this pattern may reflect elevated motor activity throughout the 24-hour cycle—including during nocturnal awakenings—resulting in more constant activity levels and fewer hour-to-hour transitions, thereby paradoxically reducing overall rhythm variability (Table 2).

In addition, we also explored the relationship between actigraphic and cognitive/behavioral characteristics, ED symptoms, psychiatric comorbidities, and autistic traits in adults with ADHD. We also compared cognitive and behavioral parameters as a function of sleep efficiency. Interestingly, longer total sleep time was associated with greater emotional hyperreactivity within the ADHD group, particularly when sleep duration was averaged across weekdays and weekends compared to weekdays only. This unexpected finding should be interpreted with caution. Rather than indicating a detrimental effect of longer sleep duration, it may reflect increased fatigue, greater sleep need, and compensatory sleep behaviors in individuals with greater ED [72]. This pattern could suggest that individuals with higher emotional hyperreactivity tend to extend their sleep on weekends, which may reflect compensatory recovery following weekday sleep restriction. The absence of a link between circadian disturbances and depression or anxious symptoms is consistent with our previous study, which showed that subjective sleep disturbances were more closely related to core ADHD traits rather than depressive symptoms [73].

Although correlations between sleep parameters (including sleep onset latency, sleep efficiency, and total sleep time) and cognitive/behavioral dimensions did not survive correction for multiple comparisons, exploratory analyses revealed suggestive patterns that warrant further investigation in larger, more homogeneous samples. Additionally, descriptive comparisons suggested that adults with ADHD with low or median sleep efficiency tended to show more pronounced difficulties in cognitive functions and behaviors related to the three core symptoms of ADHD, particularly in selective attention, verbal inhibition, and hyperactivity.

Regarding attentional and executive functions, exploratory correlations suggested that higher sleep efficiency and shorter sleep latency were associated with better selective attention scores in adults with ADHD. Shorter sleep duration was also associated with longer response times in the Hayling test. In addition, shorter total sleep time on weekdays was associated with longer responses in the Go-Nogo task. These preliminary patterns suggest potential relationships between sleep duration and inhibition performance, and between sleep efficiency/latency and selective attention in adults with ADHD, though these associations did not survive correction for multiple comparisons and require replication.

Exploratory analyses suggested that higher sleep efficiency was associated with fewer symptoms of hyperactivity in adults with ADHD. In addition, motor hyperactivity was positively associated with the duration of prolonged nighttime awakenings, particularly in individuals with the combined presentation of ADHD. This pattern may reflect a bidirectional relationship: on one hand, poor sleep may beassociated with increased motor activity [40]; on the other hand, hyperactivity itself may relate to sleep continuity. Supporting this bidirectional link, higher nocturnal activity levels (L5) were associated with reduced sleep duration and lower sleep efficiency, while greater daytime activity (M10) was linked to more stable inter-day rhythms and reduced intra-day variability. This interpretation aligns with prior studies showing that hyperactivity reduction via psychostimulants can be associated with improvement in insomnia symptoms in adults with ADHD [20, 69]. Similarly, racing thoughts, conceptualized as an internalized form of hyperactivity, was also associated with long nighttime awakenings. This finding is consistent with a study showing that patients with maintenance insomnia experience more severe racing thoughts than control subjects [74].

One limitation of this study is the lack of statistical power in the correlations between the actigraphic parameters and the cognitive and behavioural dimensions of ADHD, as none of these associations remained significant after correction for multiple comparisons. This may partly stem from the inability to reduce dimensions via PCA, given the multidomain overlap of cognitive tests. Additionally, sample heterogeneity, which included participants with psychiatric and neurodevelopmental comorbidities, further complicated the identification of specific associations. While the inclusion of comorbid condition increases ecological validity, given that over 80% of ADHD cases present with at least one comorbidity [4], it also complicates the identification of specific associations between ADHD symptoms and sleep or circadian disruption, which may also arise from the comorbid conditions themselves (e.g., mood disorders, autism; [75, 76]). Future studies should employ larger sample sizes with adequate power for multiple comparisons and consider alternative analytical approaches such as mixed-effects models or stratified analyses based on comorbidity profiles to better characterize these relationships.

Although age was corrected at a later stage, the age differences between our participants also limit the scope of our results with regard to the age-related differences commonly observed in sleep and circadian rhythm parameters [67] as well as in socio-professional categories [66]. Indeed, socio-professional status differed between groups: a higher proportion of adults with ADHD were unemployed or on disability leave, whereas most control participants were students (Table 1). Importantly, this may have attenuated the observed between-group differences in circadian phase, as the control group—being mostly students and young adults rather than employed—would likely have been more phase delayed than a similarly aged fully employed group [66, 67]. The predominantly student composition of the control group may also explain why we did not observe the expected between-group difference in sleep efficiency, as students typically exhibit poorer sleep efficiency [77].

Another limitation lies in the use of different actigraphic devices across groups, which prevented the comparison of certain sleep parameters, such as the number and average duration of awakenings [78]. Additionally, ED was measured via a trait questionnaire – i.e., WRAADDS, ALS –, which may suffer from recall biases, whereas it should probably be investigated using other experimental measures, such as emotion induction paradigms [32].

Our findings highlight a pattern of rhythm instability and phase delay in the sleep-wake rhythms in adults with ADHD from a clinical population, emphasizing the importance of incorporating sleep and circadian factors into both diagnosis and treatment strategies. Sleep quality appears closely linked to core ADHD symptoms, particularly hyperactivity, with distinct cognitive effects: longer sleep latency is associated with poorer selective attention, while shorter sleep duration correlates with deficits in inhibitory control. These results suggest that sleep disturbances may contribute to ADHD-related cognitive and behavioral symptoms, often manifesting as racing thoughts or mental restlessness. Emerging evidence further indicates that chronotherapeutic interventions — such as regularising sleep schedules, timed morning bright light exposure [79], and low-dose melatonin [80] aimed at advancing delayed circadian phase — could improve both sleep/circadian rhythms and core ADHD symptoms [81].

## Supplementary Information

Below is the link to the electronic supplementary material.

Supplementary Material A

Supplementary Material B

## Data Availability

The datasets used and/or analysed during the current study are available from the corresponding author on reasonable request.

## Abbreviations

ADHD: Attention Deficit Hyperactivity Disorder
ED: Emotion Dysregulation
REM: Rapid Eye Movement
PSQI: Pittsburgh Sleep Quality Index
PSG: Polysomnography
DSM: Diagnostic and Statistical Manual of Mental Disorders
ESS: Epworth Sleepiness Scale
ISS: Insomnia Severity Index
RLS: Restless Legs Syndrome
OSA: Obstructive Sleep Apnea
MEQ: Morningness-Eveningness Questionnaire
WRAADDS: Wender-Reimherr Adult Attention Deficit Disorder Scale
ALS: Affective Lability Scale
RCTQ: Racing and Crowded Thoughts Questionnaire
BDI: Beck Depression Inventory
GAD-7: Generalized Anxiety Disorder assessment-7-item
AQ-10: Autism Spectrum Quotient-10-item
WAIS-III: Wechsler Intelligence Scale for Adults − 3rd edition
TAP: Test of Attentional Performance
TMT: Trail Making Test
TST: Total Sleep Time
WASO: Wake-up After Sleep Onset
NPCRA: Non-Parametric Circadian Rhythm Analysis
IV: Intra-day Variability
IS: Inter-day Stability
M10: Start of most active phase (10 h)
L5: Start of least active phase (5 h)
ANCOVA: Analysis of Covariance
FDR: False Discovery Rate
TOST: Two One-Sided Test
RT: Reaction Time
TEMPS: Temperament evaluation (cyclothymic temperament)
