# Supplementary material for: Sleep, rest-activity rhythm, cognitive and emotional symptoms in adult ADHD: unraveling the links with an actimetry-based approach
Source: BMC Psychiatry. 2026 Mar 9;26:317. doi: 10.1186/s12888-026-07947-9 (PMC13085561; doi:10.1186/s12888-026-07947-9)
Supplement: Supplementary file 1 — Supplementary Material A [file 12888_2026_7947_MOESM1_ESM.docx]

**Supplementary materials**

**A. Table 1. Comparison of sleep parameters measured between Motionwatch and ActiGraph watch (n = 8)**

|  | | t | df | *p-*value | Effect Size  Hedges’g | |
| --- | --- | --- | --- | --- | --- | --- |
| **Sleep latency** | t test | - 0.525 | 7 | .616 | | −0.176 |
|  | TOST lower | - 0.334 | 7 | .626 | |  |
|  | TOST upper | - 0.716 | 7 | .249 | |  |
| **TST (min)** | t test | 0.030 | 7 | .977 | | 0.0102 |
|  | TOST lower | 0.071 | 7 | .473 | |  |
|  | TOST upper | - 0.010 | 7 | .496 | |  |
| **Sleep efficiency** | t test | 0.192 | 7 | .853 | | 0.0641 |
|  | TOST lower | 0.354 | 7 | .367 | |  |
|  | TOST upper | 0.030 | 7 | .512 | |  |
| **WASO** | t test | - 1.039 | 7 | .333 | | -0.347 |
|  | TOST Lower | - 0.973 | 7 | .819 | |  |
|  | TOST upper | - 1.105 | 7 | .153 | |  |
| **Number of awakenings** | t test | 3.08 | 7 | **.018*** | | 1.03 |
|  | TOST lower | 3.30 | 7 | **.007**** | |  |
|  | TOST upper | 2.87 | 7 | .988 | |  |
| **Mean duration of awakenings** | t test | - 2.75 | 7 | **.029*** | | -0.919 |
|  | TOST lower | - 1.68 | 7 | .932 | |  |
|  | TOST upper | - 3.82 | 7 | **.003**** | |  |

**p < 0.05; ** p < 0.01*

*TST = Total Sleep Time; WASO = Wake-up After Sleep Onset*

No significant differences were observed between devices for sleep latency, total sleep time (TST), sleep efficiency, or wake after sleep onset (WASO) (all p > 0.05), with very small to small effect sizes (Hedges’ g ≤ 0.35).

In contrast, significant differences between devices were observed for the number of awakenings (t(7) = 3.08, p = .018) and the mean duration of awakenings (t(7) = −2.75, p = .029), both associated with large effect sizes (Hedges’ g = 1.03 and −0.92, respectively).
