# Supplementary material for: Sleep, rest-activity rhythm, cognitive and emotional symptoms in adult ADHD: unraveling the links with an actimetry-based approach
Source: BMC Psychiatry. 2026 Mar 9;26:317. doi: 10.1186/s12888-026-07947-9 (PMC13085561; doi:10.1186/s12888-026-07947-9)
Supplement: Supplementary file 2 — Supplementary Material B [file 12888_2026_7947_MOESM2_ESM.docx]

**Supplementary materials**

**B. Table 2. Demographic, sleep and circadian rhythm characteristics of participants according to ADHD presentation**

|  | **ADHD inattentive***  (n = 10) | **ADHD**  **combined***  (n = 42) | *p*-value | *p-*value after adjustment | Effect size  ( η²p and ε²) |
| --- | --- | --- | --- | --- | --- |
| **Demographic characteristics** | | | | | |
| Age^a^ | 30.3 (7.79) | 33.3 (10.2) | .480 | **-** | **-** |
| Sex | n = 5 F, 5 M | n = 25 F, 17 M | .584 | **-** | **-** |
| **Sleep and circadian rhythm characteristics** | | | | | |
| Sleep latency (min)^a^ | 7.56 (2.99) | 9.54 (4.32) | .182 | .343 | .018 |
| TST (min) | 441 (68.4) | 449 (55.5) | .708 | .444 | .003 |
| Sleep efficiency^a^ | 89.8 (4.72) | 87.3 (4.72) | .134 | .141 | .043 |
| WASO (sum of awakenings ≥ 1 min) | 42.8 (25.7) | 57.2 (23.3) | **.093** | **.088** | .061 |
| WASO (sum of awakenings ≥ 5 min) | 23.8 (13.4) | 35.9 (18.9) | **.062** | **.056** | .074 |
| Inter-day stability (IS) | 0.427 (0.168) | 0.459 (0.114) | .520 | .656 | .005 |
| Intra-day variability (IV) | 0.898 (0.203) | 0.876 (0.217) | .790 | .816 | .001 |
| Start of most active phase (M10)^a^ | 15.7 (2.73) | 15.6 (2.32) | .688 | .799 | .001 |
| Start of least active phase (L5)^a^ | 3.97 (2.92) | 4.53 (1.83) | .240 | .183 | .038 |
| M10 (counts)^a^ | 2412 (596) | 2475 (851) | .839 | .713 | .003 |
| L5 (counts)^a^ | 220 (143) | 174 (135) | .183 | .258 | .028 |

*Data are presented as n or mean (SD).*

*p-values in bold < 0.10*

*^a^Non-parametric test applied to non-normal or non-homogeneous data*

*TST = Total Sleep Time; WASO = Wake-up After Sleep Onset*

**The inattentive presentation of ADHD consist of inattention symptoms and the combined presentation is characterized by inattention, hyperactivity, and impulsivity.*
